# Lessons From the Virtual Fracture Clinic: An Efficient Model With Satisfied Patients

**DOI:** 10.7759/cureus.30413

**Published:** 2022-10-18

**Authors:** Iolo Thomas-Jones, Cezary Kocialkowski, Elizabeth Dominguez, James Williams

**Affiliations:** 1 Bristol Medical School, University of Bristol, Bristol, GBR; 2 Department of Trauma and Orthopaedics, Musgrove Park Hospital, Taunton, GBR

**Keywords:** fracture clinic, british orthopaedic association, trauma and orthopaedic, low energy fracture, non-significant injury, resource re-distribution, musculoskeletal services, patient experience, patient satisfaction, virtual fracture clinic

## Abstract

Introduction

The virtual fracture clinic (VFC) can be an effective means of managing specific musculoskeletal injuries, including through protocol-driven direct discharge from the emergency department (ED) or minor injury unit (MIU). This study aimed to measure the efficiency of the VFC triage process while quantifying patient satisfaction after direct discharge from the VFC without routine face-to-face review.

Methods

We conducted a retrospective analysis of a prospectively collected database to ascertain VFC outcomes for 9064 patients from February 2017 to July 2018. We analysed consultant variation in VFC review and the mean time taken per patient. Patient satisfaction was investigated in 100 chronological patients initially managed via the VFC.

Results

The mean time to triage each patient was 74 seconds, and a mean of 503 patients was triaged each month. The telephone helpline received a mean of 0.9 calls per week. Seventy-seven percent of patients stated they were happy to be managed without a fracture clinic appointment, and 82% of patients received virtual leaflets in the ED or MIU as per protocol. The number of fracture clinic appointments was reduced by 24% as patients were discharged directly or seen instead by allied health professionals. The median patient satisfaction with VFC management was 90%, and the mean satisfaction with VFC communication was 80%.

Conclusion

Virtual review of fracture clinic referral enables stratification of patients according to clinical urgency and saves patients from attending unnecessary appointments. This is achieved in a patient population generally satisfied with their overall management. Our study results support the growing trend of VFCs as pivotal systems in streamlining the care of musculoskeletal injuries while adhering to British Orthopaedic Association Standards for Trauma guidelines for Fracture Clinic Services.

## Introduction

Variations of the virtual fracture clinic (VFC) pioneered by the Glasgow Royal Infirmary have become a common component of musculoskeletal service provision within the National Health System in the United Kingdom (UK) [1]. The VFC has been identified as a reproducible, safe, and cost-effective means of achieving good functional outcomes for patients with specific musculoskeletal injuries [2-7].

Attendances to emergency departments (EDs) have increased by 30% since 2004 and are increasing faster than the population growth [3,4]. Musculoskeletal and soft tissue injuries comprise approximately 30% of attendances, with 75% to 92% of these injuries being followed up by an orthopaedic surgeon [3,5].

In the traditional fracture clinic model, issues arise due to increasing patient numbers and costs, dependence on junior support in clinical decision-making, and regular fracture clinic delays, which likely contribute to poor patient satisfaction. Developing a new fracture clinic management system enables patient review and rationalisation of resource distribution [4,6,7]. The VFC depends on collaboration with the ED for protocol-driven and leaflet-supported direct discharge of suitable injuries. Patients are also encouraged and empowered to take responsibility for the care of their nonsignificant injury, with virtual care leaflets containing information on rehabilitation and what to do if issues arise [3,6-8].

The ED and neighbouring minor injury units (MIUs) at our institution refer fracture clinic patients to the VFC. Referrals to VFC are triaged daily by senior orthopaedic surgeons in conjunction with a trauma nurse coordinator. Patients are triaged into the following categories: direct discharge, next available general fracture clinic, specialist upper/lower limb fracture clinic, physiotherapy appointment, or an appointment with an advanced orthopaedic practitioner (AOP) for stable injuries that require simple splinting and clinical review.

Our institution uses template letters for the most common fractures amenable to conservative management (e.g., fifth metacarpal fractures, fifth metatarsal fractures, paediatric clavicle fractures, paediatric torus fractures, radial head/neck fractures, and mallet finger injuries). These letters are issued to the patient after VFC review. Patients with other minor injuries amenable for direct discharge (in addition to the aforementioned fractures managed conservatively) have a descriptive diagnosis inserted into a template letter containing generic advice on the management of a nonsignificant injury, with information on weaning out of splints and boots and what to do if pain persists. All patients receive a telephone helpline number to contact if any issues occur.

Training days were held for senior orthopaedic surgeons to standardise the patient reviewing process between clinicians before the rollout of the new fracture management system. Training days were also held for the ED and neighbouring MIUs and were supported by written protocols and guidance to support emergency physicians' decision-making concerning management and referrals. Fracture clinic referrals containing patient details and the clinical history with examination findings are referred to a single email inbox, ensuring a single point of communication between referring centres and the orthopaedic department.

The concept of the VFC aims to stratify minor injuries according to clinical need and the appropriate subspecialty or practitioner most suited to the individual injury. To date, the consequence of this patient streaming process and its effect on reducing the number of face-to-face consultations with an orthopaedic surgeon has not been well reported. Our version of the VFC devised a means to directly discharge patients without a physical review after their emergency attendance. Despite its benefits in resource management, the patient experience after direct discharge following VFC review requires investigation and scrutiny to ensure patients feel they are receiving the optimum care. Therefore, the goals of this study were to quantify the efficiency and outcomes of VFC triage at our institution and measure patient satisfaction with VFC direct discharge while ensuring our service adhered to the British Orthopaedic Association Standards for Trauma and Orthopaedics (BOAST) guidelines on fracture clinic services [9].

## Materials and methods

Study design

Following the implementation of the fracture clinic redesign and introduction of the VFC at our institution, Musgrove Park Hospital, Taunton, UK, we conducted a retrospective analysis study of prospectively collected administrative information from all patients and their outcomes from the VFC's inception in February 2017 to July 2018. Using this database, we analysed 9064 patient records. We collated the date of injury, the number of days between ED attendance and VFC review, the outcome for each patient, and the clinician responsible for each review session. The time to conduct each VFC session for each consultant was noted and divided by the number of patients triaged in each session to generate a mean time to review each patient. We could not collect or review demographic information given the anonymity of the database.

To quantify patient satisfaction with being directly discharged without face-to-face review, 100 consecutive patients discharged directly between March 2018 and July 2018 were sent postal questionnaires during July 2018. The survey questionnaire contained several graded questions on specific aspects of the service, with 0 being least satisfied to 10 being most satisfied. The survey questionnaire contained several binary 'yes' or 'no' questions. Local institutional ethical approval was sought and granted.

Inclusion and exclusion criteria

The study included all patients referred to the VFC through the designated email pathway from the ED or a local MIU from February 2017 to March 2018 whose details were included in the local database during the study period.

Quantifying patient satisfaction

The study included 100 patients managed through direct discharge who presented to the ED or a local MIU and were referred to the VFC from March 2018 to July 2018. Patients were excluded if they were triaged to all other available outcomes, including general fracture clinic, specialist fracture clinic, advanced orthopaedic practitioner or a physiotherapist. We contacted any patients via phone who did not return their postal questionnaire after 14 days in the pre-addressed, prepaid envelope. After two attempts at contacting them by phone, they were recorded as non-responders.

Data collection

Data was collated and analysed within Microsoft Excel 2016 Version 16.0 for Windows (Microsoft, Inc., Redmond, Washington, United States). Master tables were generated for the data points of interest: date of injury, number of days between ED attendance and VFC review, review outcome for each patient, and the clinician responsible for each review session. Patient satisfaction survey responses were collated manually.

Statistical analysis

We used the independent t-test for the mean number of face-to-face fracture clinic appointments in the presence and absence of VFC. We used IBM SPSS Statistics for Windows, Version 24.0 (Released 2016; IBM Corp., Armonk, New York, United States) for all analyses, and p<0.05 was considered statistically significant.

## Results

Of the total patients triaged since the implementation of the VFC, the distribution of pathways was 5277 (58%) to a general fracture clinic, 1673 (18%) to a specialist fracture clinic, 1244 (14%) to an AOP clinic, 437 (5%) to a physiotherapy clinic, and 433 (5%) discharged directly without immediate face-to-face review. The mean time to triage each patient was 74 seconds (range, 65 to 103 seconds). A mean of 503 patients per month were reviewed virtually (range, 404 to 604 patients/month), resulting in a mean of 385 patients seen in face-to-face fracture clinic appointments, reducing the mean fracture clinic numbers per month by 118 patients (p=0.00024). The mean time between ED presentation and VFC review was 1.6 days. The mean number of calls to the helpline provided was one call per week (range, zero to three calls/week).

Patients' median satisfaction rates were 90% and 80% for satisfaction with overall management and communication, respectively. Most patients (80%) were satisfied with the consistency between what they were told in the ED/MIU and the contents of their letters following the VFC outcome. Also, 90% of patients were satisfied with the information provided by the ED or MIUs, and 100% were satisfied with the information within the letter (Table 1). Twenty-three percent would have preferred a face-to-face consultation, and 23% sought further medical advice regarding their injury. Further, 18% stated they did not receive an information leaflet on the VFC from the ED or MIU, while 40% of patients stated there was no further information they would have liked to receive regarding their injury and management plan (Table 2).

**Table 1:** Patient satisfaction scores following direct discharge after VFC review Note: 45/100 patients responded. Questionnaire scored from 0 (least satisfied) to 10 (most satisfied). ED: emergency department; MIU: minor injury unit; IQR: interquartile range; VFC: virtual fracture clinic

Patient satisfaction with:	Mean	Median	IQR	Mode
Overall management	7.7	9	4	10
Overall communication	7.2	8	5	10
Consistency between ED/MIU and virtual care letter	7.2	8	5	10
Information provided by ED/MIU	7.3	9	3	10
Information in letter	8.0	10	5.5	10

**Table 2:** Additional patient feedback following direct discharge after VFC review Note: 45/100 patients responded. ED: emergency department; MIU: minor injury unit; VFC: virtual fracture clinic

Question:	Yes	No
Would you have preferred face-to-face consultation?	23%	77%
Did you seek further medical advice from a professional advice regarding your injury?	23%	77%
Is there anything else you would have liked to have been told about your injury and management plan?	40%	60%
Did you receive a letter in the ED or MIU?	82%	18%

## Discussion

The fracture clinic redesign reduced face-to-face appointments with an orthopaedic surgeon by 24% (2114/9054 patients). We found that 22% of patients sought further professional medical advice regarding their injury; however, we do not know whether this was via a phone call to the general practitioner (GP) or orthopaedic department, or as an additional visit to the ED, GP, or an alternative practitioner. Re-presentation after discharge from the VFC was studied by Vardy et al., who found that implementing the VFC did not increase ED attendance [6]. Brooksbank et al. investigated the re-attendance rates of patients managed via a direct discharge with a mallet finger injury. They demonstrated that seven of 36 patients presented to their GP regarding immobilisation and skin-related problems (e.g., blistering secondary to splinting), five of whom later attended a consultant-led specialist orthopaedic clinic [10].

Providing sufficient levels of reassurance to patients with minor injuries during their initial presentation to the ED is a challenge, especially in patients discharged directly from the VFC without scheduled face-to-face follow-up. The remaining 95% of patients received a face-to-face appointment with an orthopaedic surgeon, an advanced orthopaedic practitioner, or a physiotherapist. Clinicians should have a low threshold for face-to-face review with an orthopaedic surgeon in the event of clinical uncertainty regarding the mechanism of injury or examination findings; this was illustrated by the 76% of patients seen in a general or specialist fracture clinic. As additional pathways are implemented and ED training and confidence increase for managing minor injuries, we anticipate an increase in the number of minor injuries discharged directly from the VFC. When patients first present with their injuries, counselling them regarding what to expect from VFC process is central to managing patient expectations.

One area identified as a target for improvement was the 18% of patients who did not receive any written information on the VFC in the ED before discharge. Correctly outlining the VFC process to patients before their departure from the ED may help address the 40% of patients who stated they would have liked more information about their injury and management plan. Due to the high turnover of junior doctors who work in the ED or an MIU, regular refresher training days and comprehensive induction to underline how the VFC operates are crucial for maintaining an excellent patient experience.

The combined trauma nurse coordinator and consultant orthopaedic surgeon approach when reviewing patients in the VFC enable a review and decision time of 74 seconds per patient. Patients are subsequently seen in a general fracture clinic, specialist fracture clinic, AOP clinic, physiotherapy clinic, or directly discharged. The ability to make direct referrals to physiotherapy is a requirement laid out by the BOAST guidelines on fracture clinic services [9]. The time to review each patient is comparable to other reports in the literature, quoted elsewhere as a mean of 60 seconds [2].

The outcome of the VFC clinic at our institution is conveyed to the patient by letter, while other VFC services operate through a more resource-heavy approach by telephoning each patient. Our helpline received 14 calls in two weeks, equating to an average of 6% of patients referred to the VFC in a calendar month. The nature of these patients' injuries and VFC outcomes were not recorded. Another study reported that 25% of patients used the telephone helpline [10]. The time from the initial presentation with their injury to VFC outcome is 1.6 days, which is well within the 72 hours recommended in the BOAST guidelines [9].

Patients demonstrated good overall satisfaction with the VFC, with a median satisfaction of 90% compared to the 91% to 97% satisfaction reported in other studies [1,7,11,12]. Our system had a 100% patient satisfaction score for information provided in the letter, which was higher than the 86% to 95% satisfaction reported in earlier studies [1,7,10,11,13]. In one previous study, 28% of patients would have preferred a face-to-face session, comparable to the 23% of patients in our study [12].

There are limitations to this study. The data from the local prospectively collected administrative database were anonymised for clinical information, which prevented stratification of patient surveys based on the type of injury, gender, age, and comorbidities. The relatively poor survey response rate of 45% suggests our results are susceptible to selection bias, with patients who experienced a generally more positive or negative experience may have been more inclined to respond. Furthermore, the questionnaire lacked information regarding what further medical advice patients sought after VFC discharge; this information would have enabled a more thorough assessment of the impact of VFC on the increased workload for our ED and GP colleagues.

## Conclusions

This study aimed to measure the efficiency of the VFC triage process and quantify patient satisfaction after direct discharge following VFC review. The VFC enables timely and consistent triage of patients regardless of the clinician conducting the VFC clinic, reducing the overall number of fracture clinic appointments and enabling direct referral to allied health professional clinics. This enables direct referrals to upper or lower limb specialist clinics. The VFC can achieve good patient satisfaction levels while adhering to BOAST guidelines on fracture clinic services. Further improvements are required in counselling ED patients on their injuries and ensuring patients receive the correct information to manage expectations in the days preceding VFC review. We are aware that VFCs have become an important component in many trauma and orthopaedic departments nationwide and hope the model laid out here will be helpful to other departments in any future fracture clinic re-design.
